# Cellular signatures of immune dysregulation in inborn errors of immunity: development of a quantitative immune balance score

**DOI:** 10.3389/fimmu.2026.1735655

**Published:** 2026-03-05

**Authors:** Dilan Inan, Hacer Neslihan Bildik, Busra Ciftcier, Aysegul Akarsu, Melike Ocak, Aslihan Berra Bolat, Deniz Cagdas, Ilhan Tezcan, Sevil Oskay Halacli

**Affiliations:** 1 Division of Pediatric Immunology, Department of Basic Sciences of Pediatrics, Institute of Child’s Health, Ankara, Türkiye; 2 Division of Pediatric Immunology, Department of Pediatrics, Hacettepe University Faculty of Medicine, Ankara, Türkiye; 3 Division of Pediatric Allergy, Department of Pediatrics, Hacettepe University Faculty of Medicine, Ankara, Türkiye

**Keywords:** ctfh cells, immune cell phenotype, immune cell signature, immune dysregulation, inborn errors of immunity, regulatory B cells, regulatory T cells, Th17 Cells

## Abstract

**Background:**

Immune dysregulation, classified as distinct phenotypes within inborn errors of immunity (IEIs) and collectively known as primary immune regulatory disorders (PIRD) is increasingly recognized as a major contributor to morbidity; however, the quantitative cellular signatures identifying this state remain incompletely characterized.

**Objective:**

We aimed to describe cellular changes underlying immune dysregulation and to develop an integrative biomarker framework that links inflammatory/regulatory imbalance with genetic background and clinical phenotypes.

**Methods:**

We performed multiparametric flow cytometry and computational modeling in 39 genetically defined IEI patients (PIRD and non-PIRDs) and 17 age-matched healthy controls. Correlation networks, t-SNE, and FlowSOM clustering were applied to examine regulatory and inflammatory compartments. An immune dysregulation score (IDS) was derived as the log-ratio of inflammatory to regulatory subsets, and clinical dysregulation was quantified by a composite presence/absence score. Integrated models were evaluated using ROC, calibration, and decision curve analyses in accordance with TRIPOD recommendations.

**Results:**

Patients showed significant alterations in immune networks, with consistent reductions in FOXP3^+^ Tregs and transitional Breg cells alongside expansions of BCL6^+^, CD4^+^ ICOS^+^BCL6^+^ Tfh-like subsets, and activated CD8^+^ T cells. IDS clearly distinguished patients from controls (AUC = 0.75, 95% CI 0.60–0.90, p < 0.01) and correlated inversely with clinical z scores (r = –0.65, p = 2.0 × 10^-5^). Subgroup analyses displayed elevated IDS in patients with genetically confirmed PIRDs whereas non-PIRDs showed IDS values comparable to controls. Integrating IDS with clinical features tiered patients into three clusters that consistent with the underlying genetic causes. Composite machine-learning models modestly improved stratification of mild and moderate cases, though severe phenotypes remained heterogeneous.

**Conclusion:**

Our findings identify IDS as a novel quantitative biomarker that captures the regulatory–inflammatory imbalance underlying immune dysregulation, distinguishes PIRDs from non-PIRD IEIs, and provides a translational framework for patient stratification. IDS may inform longitudinal monitoring and guide targeted therapeutic interventions in immune dysregulation syndromes.

## Introduction

Immune dysregulation represents a pathological disequilibrium in the host’s response to self or non-self antigens, revealing as excessive inflammatory or regulatory activity. Its development is driven not only by the nature, duration, and intensity of antigenic exposure but also by environmental, dietary, and therapeutic factors. At the molecular and cellular level, immune dysregulation most often arises from genetic defects that alter immune cell number or function, thereby disturbing the balance between inflammatory and regulatory compartments. Within the spectrum of inborn errors of immunity (IEIs), such defects are classically encompassed by diseases of immune dysregulation and regulatory T-cell disorders and are collectively referred to as primary immune regulatory disorders (PIRDs) (1–6).

Clinically, patients with PIRDs exhibit a broad and heterogeneous phenotype that extends beyond the infection susceptibility traditionally associated with IEIs, encompassing autoimmunity, chronic inflammation, lymphoproliferation, and malignancy (1–4, 7–9). This overlap underscores that PIRDs are not divergent phenotypes but rather form an integral subclass within the IEI context, where infectious susceptibility and dysregulated immune responses coexist as dual hallmarks of disease (1, 2, 7–10). Importantly, PIRDs span a wide biological spectrum, ranging from hyperinflammatory states driven by impaired immune tolerance—such as FOXP3 deficiency, CTLA4 or LRBA insufficiency, and STAT3 gain-of-function mutations (11–14) to mixed phenotypes in which infection risk coexists with autoimmunity and lymphoproliferation, exemplified by activated PI3Kδ syndrome (APDS) (15). Importantly, features of immune dysregulation are not confined to individuals with genes formally categorized as PIRDs in the 2024 IUIS classification; patients carrying variants in genes such as *CORO1A* may similarly exhibit heterogeneous manifestations of dysregulation (16, 17). These observations highlight the necessity of distinguishing dysregulation-driven disease from “pure” immunodeficiency within the broader IEI spectrum.

This biological diversity emphasizes a continuum linking immune deficiency and immune dysregulation two interconnected processes reflecting impaired host defense and disrupted immune homeostasis (16). Recognizing this overlap highlights the importance of investigating how genetic perturbations reshape the equilibrium between inflammatory and regulatory immune cell subsets (17–23). Holistic approaches integrating cellular and molecular profiling with genetic data are therefore essential to capture both the mechanistic complexity and clinical heterogeneity of immune dysregulation (17–23). Ultimately, identifying multidimensional biomarkers that encapsulate these alterations may provide a comprehensive framework for understanding PIRDs and their impact on disease trajectories (24, 25).

Despite substantial progress in defining the molecular basis of IEIs, major knowledge gaps remain. Current evidence consistently demonstrates loss of regulatory subsets, expansion of inflammatory populations, and disruption of their reciprocal ratios (20–23, 26). There are several attempts to clarify immune cell signature in patients with acute or chronic infections, sepsis and monogenic inflammatory diseases by analysing trascriptome profiles and clinical immune dysregulation score on the basis of clinical symptoms and severity such as IDDA2.1 (Kaleidoscope) score in IEIs (24, 27–30). However, the rarity, biological complexity, and phenotypic diversity of IEIs hinder large-scale conclusions. Genotype–phenotype discordance and incomplete penetrance further obscure causal links, such that identical variants may produce divergent clinical trajectories even within families. Moreover, individualized diagnosis and therapy remain challenging- available clinical algorithms often fail in atypical or multisystem presentations, and laboratory diagnostics face persistent barriers-including limited access to harmonized immune-cell profiling, age-related variability in reference ranges, batch effects, and the difficulty of integrating multi-omic and clinical data. As a result, comprehensive studies that integrate genetic background, standardized immune phenotyping, and longitudinal clinical outcomes are critically needed to translate mechanistic insights into actionable, patient-specific care pathways.

To address these challenges, we performed an in-depth immunophenotypic analysis of 39 patients with genetically defined IEIs—including both PIRD and non-PIRD cases—and 17 age-matched healthy controls. By systematically characterizing inflammatory and regulatory immune subsets, we developed an Immune Dysregulation Score (IDS) that integrates cellular, genetic, and clinical dimensions. This quantitative framework was designed to capture the balance between regulatory and inflammatory compartments in a biologically interpretable manner and to explore its capacity to distinguish dysregulation-driven phenotypes within the IEI spectrum. Rather than serving as an immediate clinical decision tool, IDS provides a structured, data-driven approach for immune-based patient stratification and hypothesis generation, laying the groundwork for future validation and clinical translation.

## Materials and methods

### Patients

Individuals presenting to our clinic between 2020 and 2023 with genetically defined patients with IEI were eligible for enrollment. Demographic data and clinical history of the patients, including autoimmunity, chronic inflammation, lymphoproliferation, infection, and malignancy, was retrieved from the hospital medical record system (**Supplementary Table 1**). All participants or their custodians provided written informed consent prior to study inclusion.

Age-matched healthy controls were involved from volunteers with no history of active infection, allergic disease, chronic medication use, or smoking. These exclusion criteria were confirmed by detailed history and physical examination, and written informed consent was obtained from all healthy donors.

Given the overlap of the study period with the COVID-19 pandemic, both patients and controls were additionally screened through the national health registry and by clinical evaluation for evidence of active or recent SARS-CoV-2 infection prior to inclusion.

The study protocol was reviewed and approved by the Hacettepe University Non-Interventional Clinical Research Ethics Committee (approval number GO 18/618-27). All procedures performed in studies involving human participants were conducted in accordance with the ethical standards of the institutional research committee and with the 1964 Helsinki Declaration and its later revisions or comparable ethical standards.

### Genetic background in the patients

The genetic diagnoses of all patients had been previously established as part of routine clinical evaluation. Variants were identified by standard genetic testing approaches, including targeted gene sequencing, whole-exome sequencing, and/or confirmatory Sanger sequencing, depending on the clinical rationale. All included patients carried pathogenic or likely pathogenic variants associated with PIRDs and non-PIRDs. No additional genetic analyses were performed within the scope of this study, and only individuals with genetically confirmed diagnoses were involved.

### Flow cytometry

Peripheral blood mononuclear cells (PBMCs) were isolated from freshly collected peripheral blood samples using density gradient centrifugation. Cells were then stained with pre-titrated antibody panels designed to characterize inflammatory and regulatory immune cell subsets (panels and antibody details are provided in **Supplementary Table 2**).

Data acquisition was performed on a BD FACSCanto II flow cytometer (BD Biosciences, San Jose, CA), and compensation controls were included for each run. Flow cytometric data were analyzed using FlowJo software, version 10.0 (TreeStar Inc., Ashland, OR). Gating strategies were applied to identify and measure both inflammatory and regulatory subsets (**Supplementary Figure 1**). Results were expressed as percentages of parent populations, as appropriate.

### Statistical analysis

All statistical analyses were performed using R (version 4.5.0, R Foundation for Statistical Computing, Vienna, Austria) and Python (version 3.11, Python Software Foundation).

To explore cellular perturbations, cross-correlation matrices were create separately for patients and healthy controls, followed by delta-correlation network analysis to identify transitions in relationships between inflammatory and regulatory subsets. Dimensionality reduction was performed using t-distributed stochastic neighbor embedding (t-SNE, R package Rtsne) to visualize clustering differences between groups. For each immune subset, group comparisons were carried out using Mann–Whitney U or Wilcoxon rank-sum tests, with false discovery rate (FDR) correction applied to adjust for multiple testing.

Metacluster characterization and comparison between patients and controls were performed using unsupervised clustering (FlowSOM, R package FlowSOM), and significance was assessed with FDR-adjusted p-values. To better capture the directional differences in metacluster distributions between patients and controls, we performed a composition-based comparative analysis of metacluster proportions. Group-level differences were summarized and visualized using forest plots with 95% confidence intervals, allowing an intuitive assessment of both the direction and magnitude of changes across MC1–MC8. This approach was used as an exploratory, descriptive framework to support the interpretation of the clustering results. The discriminative power of significant markers was evaluated using receiver operating characteristic (ROC) curve analysis (pROC package), with the area under the curve (AUC) reported. Optimal threshold was determined by Youden index.

Heatmaps (pheatmap package) were used to illustrate cellular distribution within metaclusters, while alluvial plots (ggalluvial package) were generated to depict cellular and clinical cluster transitions.

To quantify immune dysregulation, an immune dysregulation score (IDS) was calculated by referencing the balance of pro- and anti-inflammatory subsets in patients against healthy controls. Overview of calculation of the IDS was given in **Supplementary Table 3**. IDS was calculated as shown in below:

IDS = log (geometric mean [BCL6^+^, ICOS^+^BCL6^+^, CD8^+^CD25^+^]/geometric mean [CD4^+^CD25^hi^ CD127^lo^FOXP3^+^, transitional Breg, memory Breg]).

Ratios of inflammatory to regulatory cell populations were also computed relative to controls. For clinical features, multidimensional scaling (MDS, vegan package) using Jaccard distance was applied to define clinical clusters, which were subsequently integrated with genetic backgrounds through alluvial visualization.

Clinical dysregulation scoring (CDS) was performed by systematically evaluating the presence or absence of predefined clinical domains, including autoimmune/inflammatory features, malignancy, lymphadenopathy, recurrent infections, hypogammaglobulinemia/hypergammaglobulinemia, and hepatosplenomegaly. Each variable was coded as a binary parameter (0 = absent, 1 = present) for every patient. Scores were calculated only for patients with available clinical data; thus, clinical scoring analyses were conducted in 36 patients, whereas immunological analyses included the full cohort (39 patients and 17 healthy controls).

Associations between IDS scores and CDS were tested using correlation analyses, and significant relationships identified by the selbal compositional balance approach (selbal package). Integrated analyses of immune and clinical IDS stratified patients into severity-based clusters (mild, moderate, severe).

To evaluate the predictive capacity of these features, we constructed composite models incorporating four parameters (Pro_mean, Anti_mean, IDS, delta_IDS). Both a Random Forest classifier (randomForest package) and a multinomial logistic regression (one-vs-rest) model (nnet, caret) were trained to classify patients into mild, moderate, or severe stages. Model discrimination was assessed using multiclass ROC analyses (pROC), calibration was tested by decile-binned calibration curves (rms), and clinical utility was quantified by decision curve analysis (DCA) (rmda).

Model development and reporting adhered to the TRIPOD recommendations for multivariable prediction models. Discrimination was assessed using one-vs-rest ROC curves with AUC and 95% CIs; calibration was evaluated with decile-binned calibration plots using the rms package (including calibration intercept and slope), and clinical utility with decision curve analysis (rmda). Internal validation was performed via bootstrap resampling (≥500 iterations) to obtain optimism-corrected estimates of discrimination and calibration; results were concordant in a sensitivity analysis using repeated 10-fold cross-validation.

All Figures and statistical outputs were generated using ggplot2, pheatmap, ggalluvial, pROC, FlowSOM, Rtsne, selbal, vegan, randomForest, caret, rms, rmda, and Python libraries pandas, numpy, matplotlib, seaborn. A two-tailed p-value < 0.05 after FDR correction was considered statistically significant.

## Results

### Correlation analyses identify perturbed immune subsets in patients

**Figure 1:**
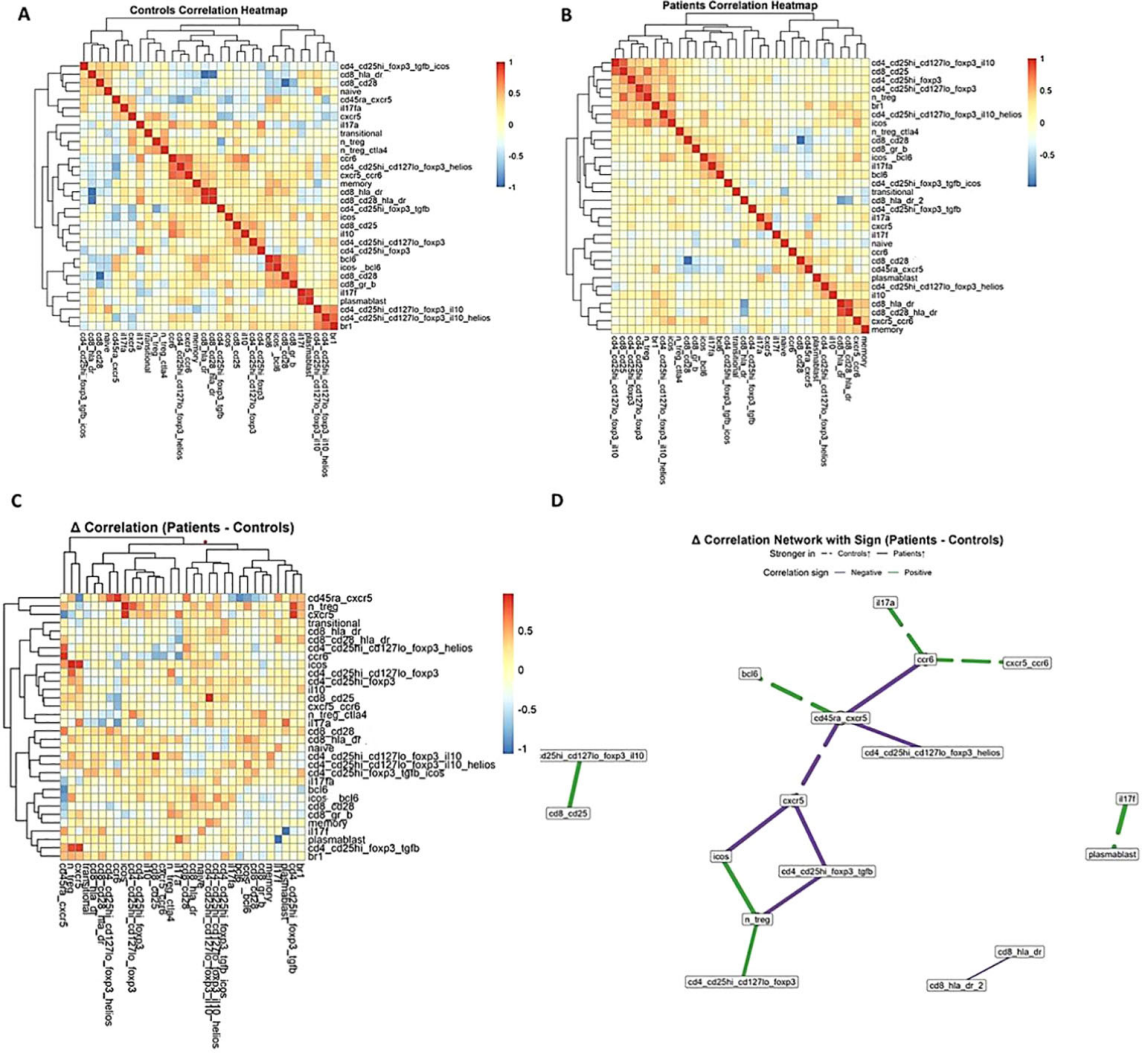
Correlation analyses reveal perturbed immune networks in patients. **(A)** Heatmap of pairwise correlations among immune subsets in healthy controls, demonstrating preserved co-regulation between regulatory and effector populations. **(B)** Heatmap of pairwise correlations in patients, showing disrupted correlation structure compared with controls. **(C)** Δ-correlation heatmap (patients – controls), highlighting immune subsets with altered correlation strength. **(D)** Δ-correlation network illustrating positive (green) and negative (purple) associations that were stronger in patients (solid line) or controls (dashed line). Notably, regulatory T cell subsets lost connectivity, whereas pro-inflammatory populations such as IL-17A+ and CCR6+ cells showed reinforced interactions.

To investigate immune-cell interactions, correlation matrices were constructed separately for healthy controls and patients. In healthy controls, regulatory and inflammatory subsets formed tightly coordinated networks, with strong positive correlations among FOXP3^+^ regulatory T-cell subsets, ICOS^+^ T cells, and CXCR5^+^ follicular helper populations. In contrast, this coordinated architecture was markedly disrupted in patients, with several of these associations weakened, lost, or reversed (**Figures 1A, B**).

Delta-correlation analyses further implicated the imbalance in the patients. Regulatory T-cell subsets (CD4^+^CD25^hi^CD127^lo^FOXP3^+^, including IL-10^+^ and Helios^+^ populations) showed notably reduced interconnectivity and diminished associations with follicular helper markers such as CXCR5 and ICOS. In contrast, inflammatory populations including IL-17A^+^ T cells, CCR6^+^ subset, activated CD8^+^ T cells (HLA-DR^+^), and plasmablasts showed enhanced positive correlations, suggesting synchronized expansion of inflammatory networks (**Figure 1C**).

Network visualization revealed a clear topological shift. Whereas healthy controls were characterized by dense regulatory connectivity, patient networks demonstrated fragmentation of regulatory nodes and the emergence of inflammatory hubs exerting dominant control over network structure (**Figure 1D**). These findings indicate that immune dysregulation in IEIs reflects not only quantitative changes in specific subsets but also a profound rewiring of cellular relationships.

### Altered cellular frequencies in patients

**Figure 2:**
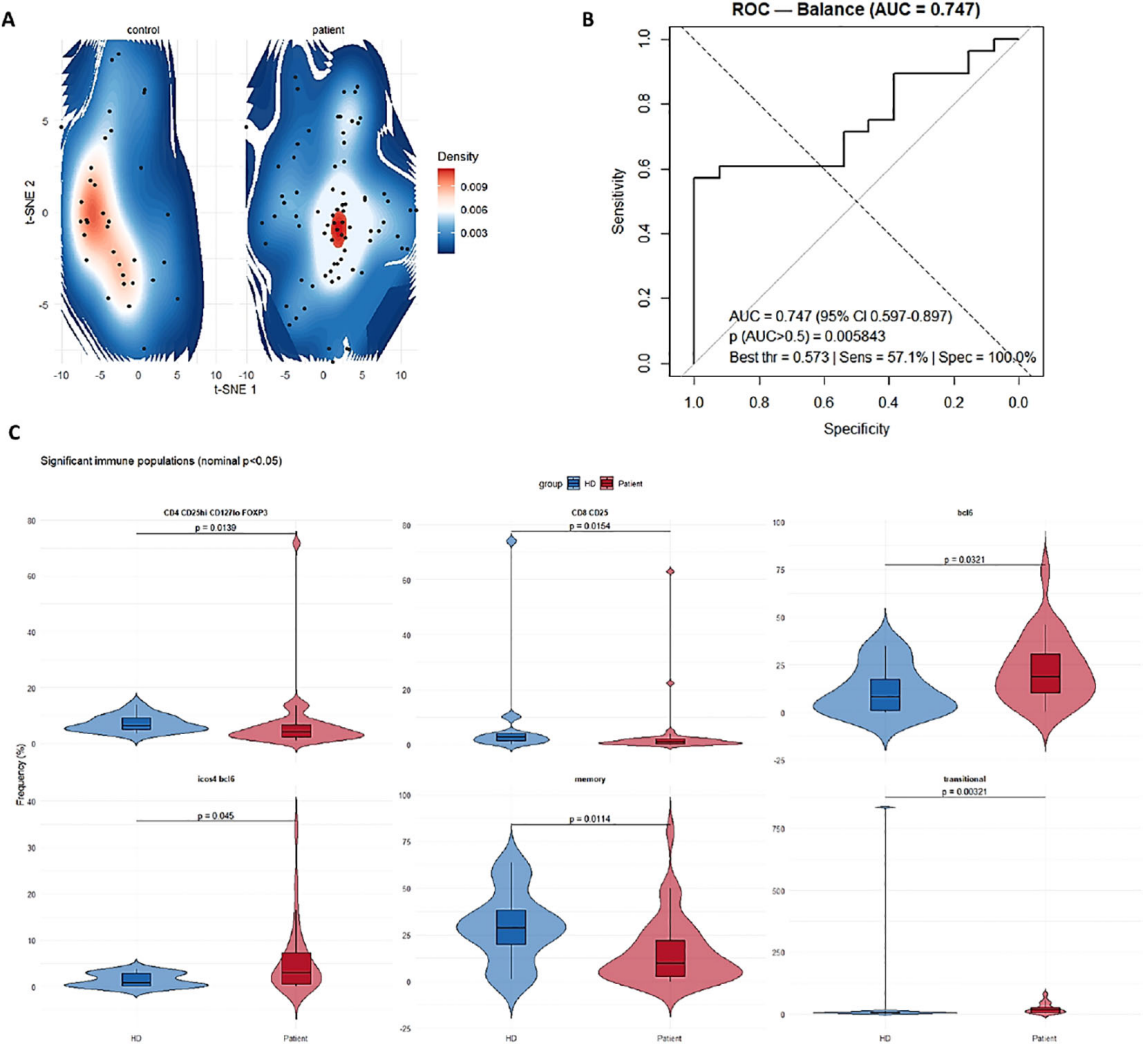
Cellular alterations and discriminative capacity of the Immune Dysregulation Score (IDS). **(A)***t*-SNE plots of peripheral immune cell subsets in healthy controls (left) and patients with immune dysregulation (right). Control samples show a broad, heterogeneous distribution, whereas patient samples form a compact and convergent pattern, indicating contraction toward a shared inflammatory profile. **(B)** Receiver operating characteristic (ROC) curve of the IDS demonstrates robust discrimination between patients and healthy controls (AUC = 0.747, 95% CI 0.597–0.897, *p* = 0.0058). The optimal threshold (0.573) yielded a sensitivity of 57.1% and a specificity of 100%. **(C)** Violin plots depicting immune cell populations that differed significantly between groups (nominal *p* < 0.05). Patients exhibited decreased frequencies of FOXP3^+^ regulatory T cells (CD4^+^CD25^hiCD127^loFOXP3^+^) and transitional B cells, together with increased frequencies of activated CD8^+^CD25^+^ T cells, ICOS^+^BCL6^+^ Tfh-like subsets, and reduced memory T-cell populations. These alterations collectively reflect an imbalance favoring pro-inflammatory over regulatory compartments.

Group-level comparisons revealed significant alterations in several populations. Frequencies of CD4^+^CD25^hi^CD127^lo^FOXP3^+^ regulatory T cells were significantly reduced in patients (p = 0.0139), whereas CD8^+^CD25^+^ T cells were expanded (p = 0.016). Inflammatory-associated populations including BCL6^+^ Tfh-like subsets (p = 0.0321) and ICOS^+^BCL6^+^ cells (p = 0.045) were increased, while memory T cells were decreased (p = 0.0114). Transitional Breg cell populations were also markedly reduced (p = 0.0032). These findings are illustrated in violin plots (**Figure 2C**).

Dimensionality reduction using t-SNE revealed distinct clustering of patients and healthy controls. Despite genetic and clinical heterogeneity, patient samples converged toward a compact immune landscape, in contrast to the broader and more heterogeneous distribution observed in controls (**Figure 2A**), indicating a shared inflammatory signature across IEIs.

### Development and performance of the immune dysregulation score (IDS)

Based on these consistent alterations, we sought to derive a quantitative measure capturing immune imbalance. Univariate analyses identified six immune subsets significantly associated with clinical severity (p < 0.05), which were integrated into the Immune Dysregulation Score (IDS). IDS was defined as the log-ratio of the geometric mean of inflammatory markers (activated CD8^+^CD25^+^ T cells, BCL6^+^, ICOS^+^BCL6^+^ T cells), to regulatory markers (FOXP3^+^ regulatory T cells, transitional Bregs and memory Bregs), thereby reflecting the net balance between inflammatory and regulatory compartments.

To evaluate the discriminatory capacity of these cellular alterations, we performed ROC analysis on the balance between pro- and anti-inflammatory subsets. The resulting IDS demonstrated robust discriminative power with an AUC of 0.747 (95% CI 0.597–0.897, p = 0.0058), confirming its potential as a biomarker to distinguish patients from controls (**Figure 2B**).

### Unsupervised metacluster analysis reveals loss of regulatory states

**Figure 3:**
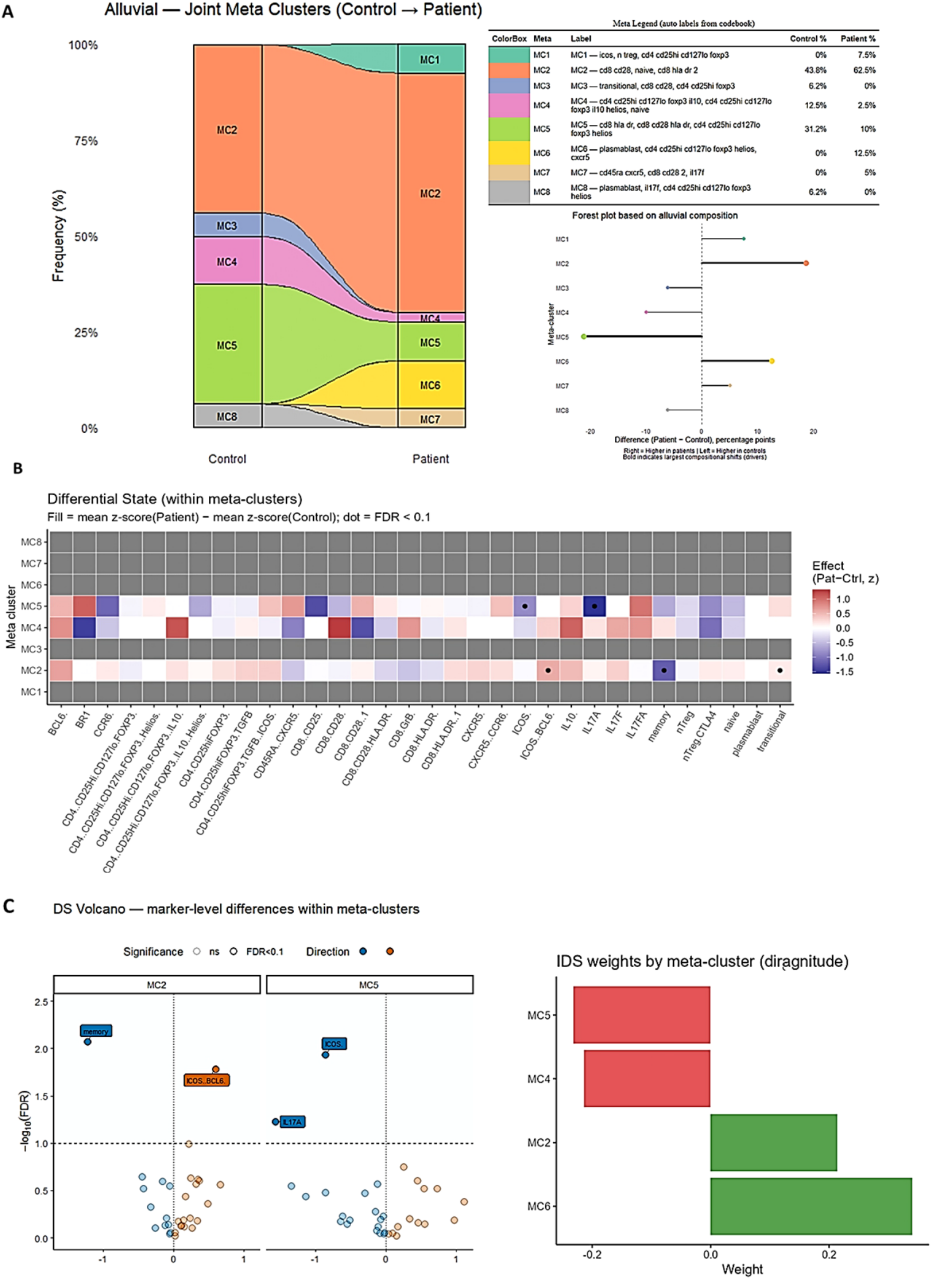
Metacluster redistribution and differential cellular states defining the IDS. **(A)** Alluvial plot showing redistribution of FlowSOM-derived metaclusters between healthy controls and patients. Eight metaclusters (MC1–MC8) were identified, encompassing distinct regulatory and inflammatory immune populations. Healthy controls were characterized by enrichment of MC2 (CD8^+^CD28^+^ naïve T cells), MC4 (FOXP3^+^ regulatory subsets), and MC5 (Helios^+^ regulatory T cells), reflecting a balanced immune architecture. In contrast, patients demonstrated expansion of MC2 (62.5%) and MC6 (plasmablast- and CXCR5-associated clusters, 12.5%), while regulatory MC4 populations markedly declined (12.5% → 2.5%) and the transitional B-cell cluster MC3 was absent. Forest plot showing the differences in FlowSOM-derived metacluster composition between patients and healthy controls based on alluvial analysis. The x-axis represents the percentage point difference (patient − control) for each metacluster (MC1–MC8). Values to the right indicate higher proportions in patients, whereas values to the left indicate higher proportions in controls. Bold lines highlight the metaclusters with the largest compositional shifts, identifying the main drivers of group differences. **(B)** Heatmap depicting differential states within metaclusters, represented as z-score differences (Patient–Control) across key markers. Patients exhibited enrichment of inflammatory features (e.g., IL-17A, ICOS, and BCL6 expression) within MC2 and MC5, together with loss of regulatory markers (FOXP3, Helios) across MC4, highlighting a pro-inflammatory shift in network topology. **(C)** Volcano plots showing marker-level differences within representative metaclusters (MC2 and MC5) indicate significant upregulation of ICOS, BCL6, and IL-17A (*FDR* < 0.1). The accompanying bar chart summarizes IDS feature weights derived from metaclusters, demonstrating dominant contributions of inflammatory clusters (MC2, MC5) and negative weights for regulatory compartments (MC4, MC6), collectively defining the cellular basis of the Immune Dysregulation Score.

To capture the intrinsic structure of immune dysregulation without imposing predefined clinical labels, we applied unsupervised clustering in comparison with healthy controls, which revealed altered cellular frequencies and convergent immune landscapes in patients. Unsupervised clustering showed eight major metaclusters (MC1–MC8) comprasing distinct inflammatory and regulatory cell subsets. Healthy controls were distinguished with MC2 (CD8^+^CD28^+^ and naive T cells, 43.8%), MC4 (regulatory FOXP3^+^ subsets, 12.5%), and MC5 (CD8^+^HLA-DR^+^ and Helios^+^ T cells, 31.2%), underpinning a balanced immune architecture. Patients, in contrast, demonstrated redistribution with expansion of MC2 (62.5%), rise of MC1 (ICOS^+^ and FOXP3^+^ Tregs, 7.5%) and MC6 (plasmablast- and CXCR5-associated cluster, 12.5%), while MC3 (transitional B cells, 6.2% in controls) was completely missing. Regulatory clusters, including MC4, were markedly reduced (12.5% → 2.5%). Alluvial visualization displayed this skewed toward effector- and inflammation-centered profiles in patients (**Figure 3A**). To further facilitate the interpretation of the clustering results, we complemented the comparison analyses with a forest plot summarizing metacluster-level differences between patients and controls based on their alluvial composition. Using composition-based comparisons of metacluster proportions, we summarized group-level differences and displayed them in a forest plot with 95% confidence intervals, enabling a straightforward evaluation of both the direction and magnitude of changes across MC1–MC8. This approach showed a consistent expansion of MC2 in patients, while MC4 and MC5 were relatively reduced. Taken together, these results offer a clearer and more intuitive quantitative view of the clustering patterns and support the presence of a shift toward reduced immune regulation in the patient group (**Figure 3A**). Heatmap and volcano plot analyses revealed enrichment of ICOS+ and BCL6+ cells within MC2 and IL-17A in MC5, concurrent with weakened memory signatures (**Figures 3B, C**). Together, these results demonstrate that IDS is characterized by both quantitative reconfiguration and qualitative state changes, with loss of regulatory T and B cell subsets and emergence of inflammatory/plasmablast-associated compartments.

### Clinical dysregulation scoring (CDS) and severity stratification

Clinical dysregulation was assessed based on the presence or absence of major immune dysregulation manifestations, including autoimmunity, chronic inflammation, lymphoproliferation, and malignancy. Each manifestation was scored as 0 (absent) or 1 (present). The individual domain scores were then summed to generate a composite clinical dysregulation score (range 0–4), where higher values indicate the coexistence of multiple dysregulation features. To clarify which clinical variables drove the clustering, we quantified the contribution of each binary feature to the k-means partitioning using variable importance analysis. The top three factors with the highest weight in determining the cluster boundaries were: (1) autoimmune/inflammatory findings, (2) hepatosplenomegaly, and (3) lymphadenopathy. These variables showed the strongest separation between the mild, moderate, and severe groups. The mild cluster was primarily driven by the absence of autoimmune/inflammatory findings, lack of lymphadenopathy or hepatosplenomegaly, and preserved immunoglobulin levels. The moderate cluster represented a heterogeneous group in which patients showed a variable combination of the assessed clinical features, without a dominant pattern driving the cluster. In contrast, the severe cluster was defined by the co-occurrence of multiple clinical abnormalities including autoimmune/inflammatory manifestations, lymphadenopathy, hepatosplenomegaly, recurrent or severe infections, hypogammaglobulinemia, and in some cases malignancy. In the binary (present/absent) clustering framework, the simultaneous presence of several of these clinical features acted as the strongest determinants distinguishing this cluster from the others.

### Integration of immune, clinical, and genetic dimensions

Having characterized disease heterogeneity using both the immune phenotyping score and the clinical dysregulation score, we sought to integrate immune, clinical, and genetic dimensions to achieve a unified, multidimensional representation of disease severity. Multidimensional scaling of clinical features stratified patients into three clusters corresponding to mild, moderate, and severe disease.

- Cluster 1 (n = 24): broad involvement with frequent autoimmunity and lymphoproliferation (severe).
- Cluster 2 (n = 7): mixed features with overlapping autoimmunity and immunodeficiency (moderate).
- Cluster 3 (n = 5): restricted phenotypes dominated by infection susceptibility (mild).

**Figure 4:**
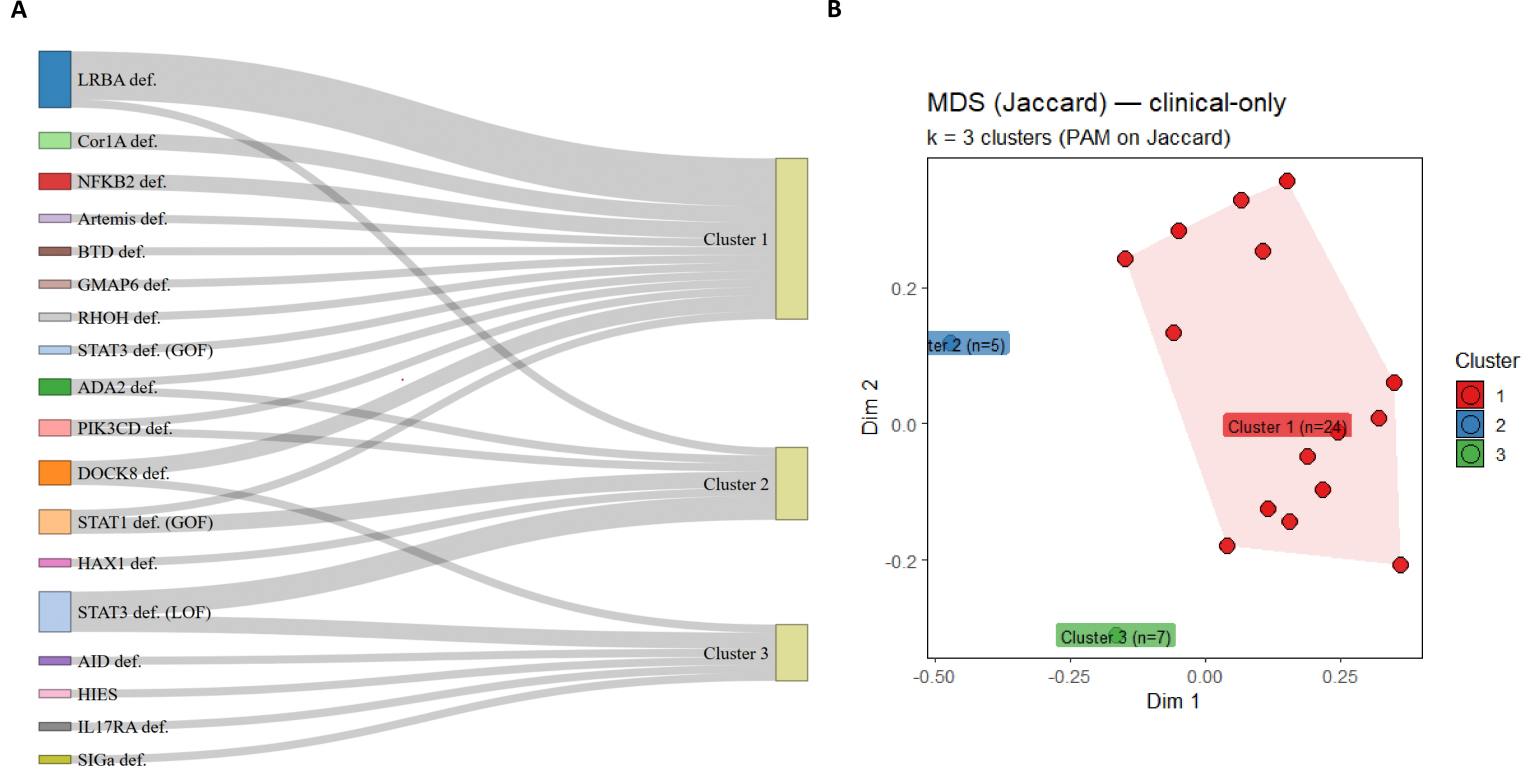
Clinical clustering and genotype–phenotype integration in patients with inborn errors of immunity (IEIs). **(A)** Alluvial diagram illustrating the association between genetic etiologies and clinical clusters identified by unsupervised analysis. Each ribbon connects a specific genetic defect to its corresponding clinical cluster. Cluster 1 (n = 24) was dominated by LRBA, STAT1-GOF, and STAT3-GOF deficiencies and was characterized by extensive immune dysregulation with autoimmunity and lymphoproliferation. Cluster 2 (n = 7) included DOCK8 and ADA2 deficiencies, representing mixed phenotypes with overlapping features of infection susceptibility and immune dysregulation. Cluster 3 (n = 5) comprised patients with STAT3-LOF, HAX1, and related defects showing infection-predominant, milder clinical presentations. **(B)** Multidimensional scaling (MDS) based on Jaccard distance demonstrates the segregation of patients into three discrete clinical clusters (k = 3, PAM on Jaccard). Cluster 1 (red) represents the most dysregulated group, Cluster 2 (blue) corresponds to mixed phenotypes, and Cluster 3 (green) to infection-dominant profiles. The distinct spatial separation of clusters reflects graded clinical heterogeneity consistent with underlying molecular mechanisms.

Integration with genetic etiologies revealed genotype–phenotype associations. LRBA deficiency, STAT1-GOF and STAT3-GOF mapped predominantly to Cluster 1 (severe), DOCK8 deficiency were enriched in Cluster 2 (moderate), while STAT3-LOF, HAX1, and ADA2 deficiency appeared in Cluster 3 (mild) (**Figures 4A, B**).

### IDS aligns with clinical severity and distinguishes PIRD phenotypes

**Figure 5:**
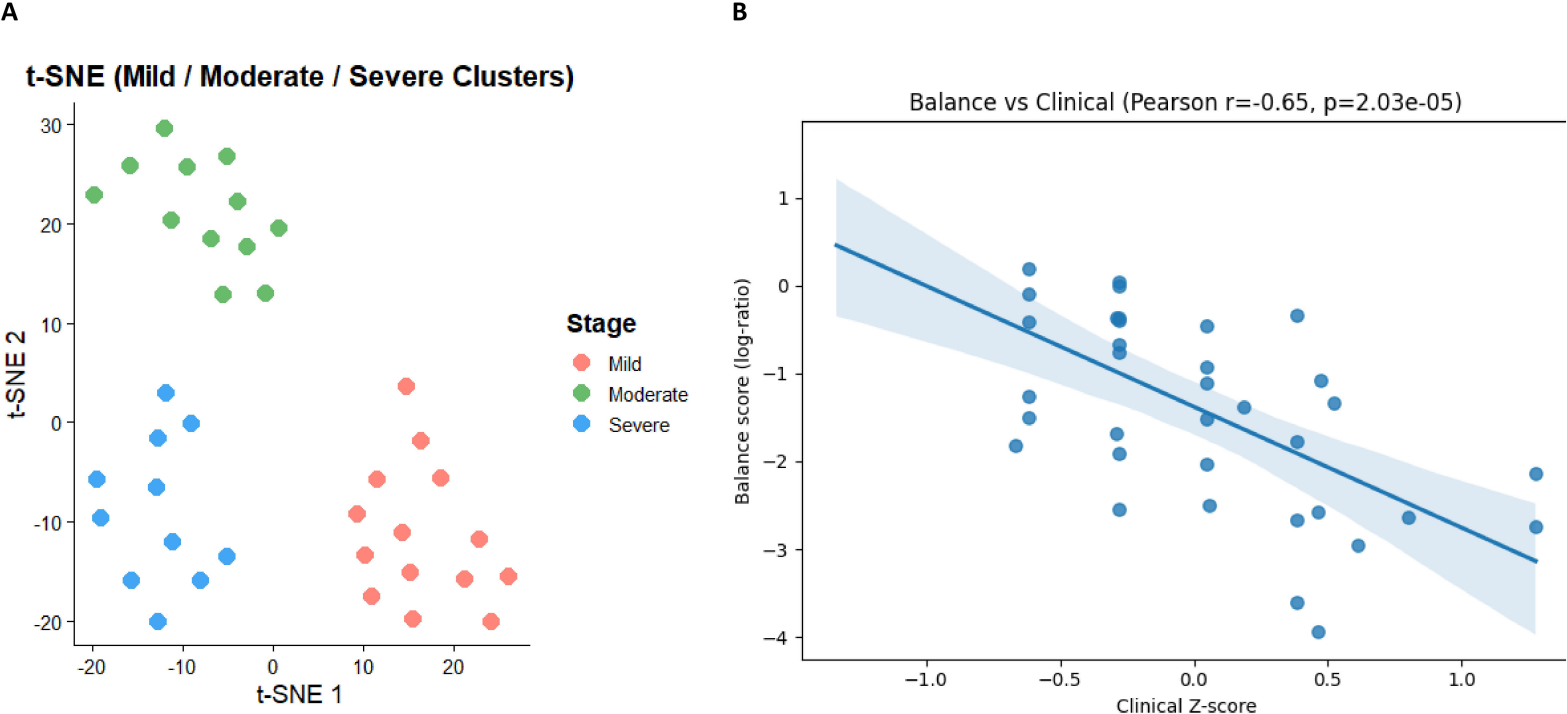
Integration of immune dysregulation and clinical severity. **(A)***t*-SNE visualization integrating Immune Dysregulation Score (IDS) with clinical features identifies three discrete patient clusters corresponding to mild (red), moderate (green), and severe (blue) disease stages. The clear spatial segregation of samples indicates that combined cellular and clinical features capture clinically meaningful heterogeneity. **(B)** Correlation analysis between immune balance score (log-ratio of inflammatory to regulatory subsets) and the composite clinical *z*-score demonstrates a significant inverse relationship (Pearson *r* = –0.65, *p* = 2.03 × 10^-5^). Increasing clinical severity is thus associated with progressive dominance of inflammatory over regulatory compartments, highlighting the quantitative link between cellular imbalance and clinical dysregulation.

To improve the integration between biological findings and computational modeling, we next examined whether the IDS reflects clinical heterogeneity across the cohort. t-SNE analysis integrating IDS with the CDS revealed three discrete patient clusters corresponding to mild, moderate, and severe disease phenotypes (**Figure 5A**), indicating that clinical heterogeneity can be captured when immune balance metrics are analyzed in conjunction with clinical data. Consistent with this clustering, the balance score showed a strong negative correlation with the clinical composite z-score (Pearson r = –0.65, p = 2.03 × 10^-5^; **Figure 5B**), highlighting a progressive shift toward inflammatory dominance with increasing disease severity.

Crucially, when we analyzed clinically defined subgroups, a clear distinction emerged. Patients fulfilling the criteria for primary immune regulatory disorders (PIRDs) showed markedly higher IDS values, whereas non-PIRD IEI patients had IDS levels comparable to healthy controls. This finding underscores that IDS is not simply a marker of immunodeficiency, but rather a quantitative reflection of immune dysregulation itself.

Taken together, these results bridge the gap between cellular immune alterations, computational modeling, and real-world clinical phenotypes. They show that IDS provides an integrated and clinically meaningful measure of immune imbalance that helps explain both disease severity and phenotypic heterogeneity across the IEI spectrum.

### Composite score analyses

**Figure 6:**
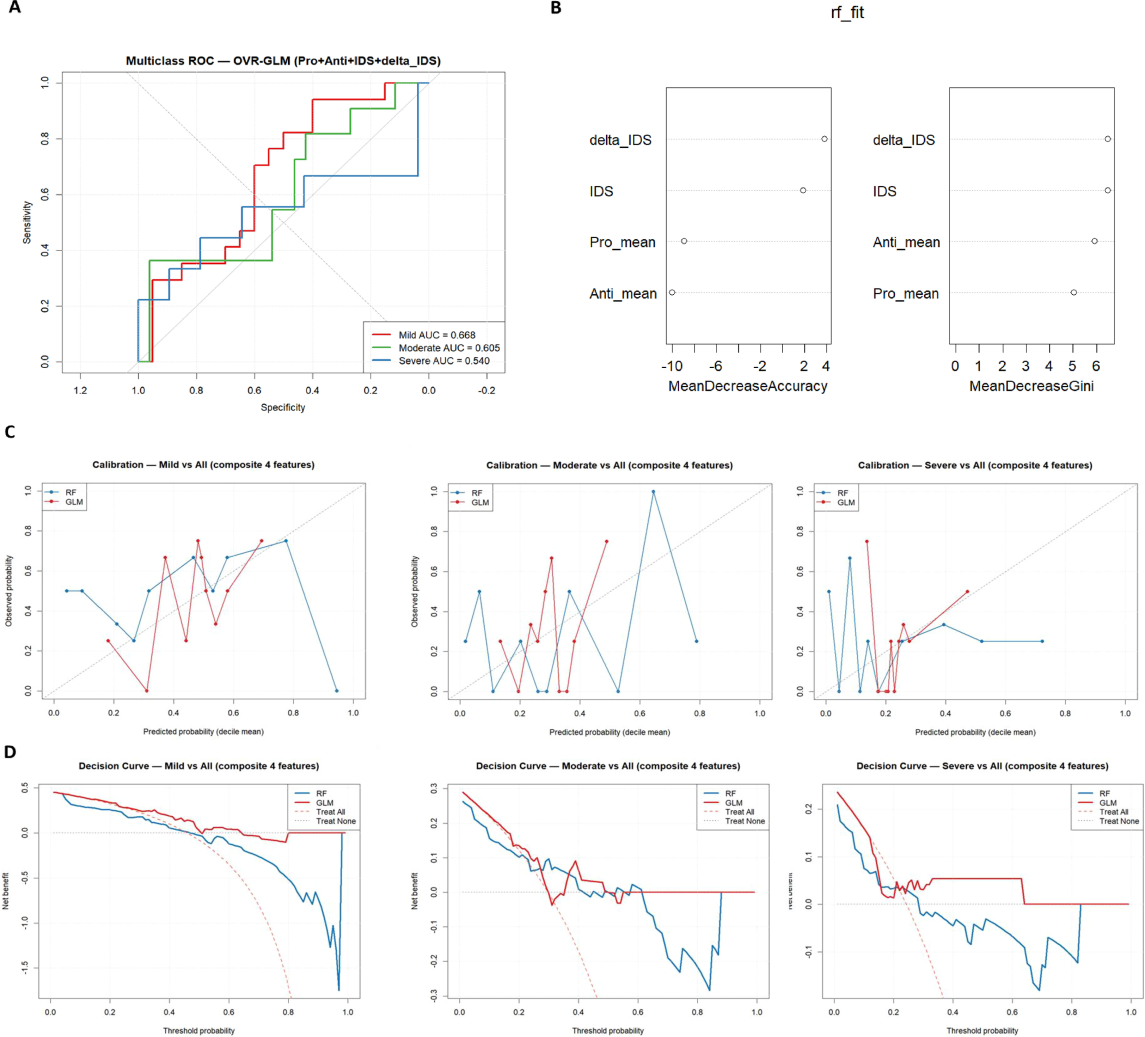
Composite model performance integrating inflammatory and regulatory features. Receiver operating characteristic (ROC), feature importance, calibration, and decision curve analyses evaluating the composite model incorporating four parameters (Pro_mean, Anti_mean, IDS, and ΔIDS). **(A)** Multiclass ROC curves (one-vs-rest GLM) demonstrate moderate discrimination for mild (AUC = 0.668) and moderate (AUC = 0.605) clinical severity groups, while severe cases remain heterogeneous (AUC = 0.540). **(B)** Random Forest (RF) variable importance plots based on Mean Decrease Accuracy and Mean Decrease Gini indicate that ΔIDS and IDS contribute most strongly to model discrimination. **(C)** Calibration plots comparing predicted and observed probabilities for mild, moderate, and severe groups show acceptable alignment for GLM at lower risk thresholds, while RF models exhibit greater variability and overestimation at higher probabilities. **(D)** Decision curve analyses (DCA) indicate modest net clinical benefit for GLM over RF across low-to-intermediate threshold probabilities, suggesting potential utility of balance-based composite metrics in stratifying clinical severity.

Although IDS alone provided clear separation of patients from controls, its discriminatory capacity across severity groups was limited. To improve this, we developed a composite model integrating Inflammatory_mean, Regulatory_mean, IDS, and ΔIDS. Feature importance analysis in RF highlighted IDS and ΔIDS as the strongest predictors. ROC analyses confirmed that the composite model achieved modest improvements in distinguishing mild and moderate groups, although severe cases remained poorly classified due to underlying heterogeneity. Calibration and DCA analyses further indicated that GLM models provided more stable estimates and modest net benefit, whereas RF tended to overestimate risk and showed variable performance (**Figures 6A–C**).

## Discussion

In this study, we extensively characterized the immunological phenotype of patients with inborn errors of immunity (IEIs) using multiparametric flow cytometry and advanced computational modeling. Our analyses demonstrated significant alterations in immune cell networks, highlighting the imbalance between inflammatory and regulatory compartments as a hallmark of immune dysregulation (18–23, 26). Importantly, our cohort included both IEI patients complying with the criteria for primary immune regulatory disorders (PIRDs) and IEI patients without apparent dysregulation phenotypes. IDS was markedly elevated in patients with PIRDs, while remaining low in non-PIRD IEI cases. This finding demonstrates that IDS specifically characterizes the immunological imbalance characteristic of dysregulation rather than reflecting immune deficiency intrinsically, thereby distinguishing clinically relevant subgroups within the heterogeneous IEI spectrum.

At the cellular level, we consistently observed decrease in FOXP3^+^ regulatory T cells and transitional Breg cells, in parallel with BCL6^+^, ICOS^+^BCL6^+^ Tfh-like subsets, and activated CD8^+^ T cells. These changes reflect those described in IPEX disease with FOXP3 mutations, CTLA4 and LRBA deficiency, and STAT3 gain-of-function disease, where disruption of immune tolerance promotes excessive inflammatory cell expansion (16–19). That the 2025 Nobel Prize in Physiology or Medicine was awarded for the discovery of FOXP3^+^ regulatory T cells emphasizes their main role in immune tolerance. In light of this finding, the substantial alterations that we observed in FOXP3^+^ Tregs (CD4^+^ CD127^lo^CD25^hi^ FOXP3^+^) provide timely and convincing evidence that disruptions in this compartment are central to the pathogenesis of immune dysregulation. In addition, in our previous study, FOXP3 expression was found to be significantly decreased in patients with autoimmune/inflammatory diseases showed diagnostic potential (21). In our current study, significantly decreased level of transitional Breg cells were determined as a discriminative marker in our cohort. This result is compatible with a study stressing significant decrease in patients with PIRDs such as LRBA and non-LRBA deficient patients (18). Similar profiles have also been reported in CVID and hypomorphic RAG deficiencies, where impaired regulation coexists with exaggerated effector responses (25, 31). Our network analyses further revealed stratification of regulatory hubs and strengthening of inflammatory nodes, paralleling previous descriptions of disrupted immune network interactions in autoimmunity and inborn errors of immunity.

To capture these perturbations quantitatively, we established an immune dysregulation score (IDS) integrating six discriminatory markers spanning regulatory and inflammatory compartments. IDS effectively separated patients from controls and correlated with clinical severity, supporting its value as a reflective measure of immune homeostasis (31). This conceptual basis supports prior concepts in which inflammatory/regulatory ratios (e.g., Th17/Treg, IL-6/IL-10) were associated with disease activity across autoimmune and inflammatory disorders (32, 33).

Our clustering results closely align with the major clinical domains repeatedly described across PIRD cohorts. Previous studies of CTLA-4 haploinsufficiency, LRBA deficiency, and related immune dysregulation syndromes consistently highlight three dominant phenotypic axes: (i) multi-organ autoimmunity and inflammation, (ii) lymphoproliferation with lymphadenopathy and hepatosplenomegaly, and (iii) immune deficiency characterized by hypogammaglobulinemia and recurrent infections, with malignancy reported in a subset of patients. Our data-driven mild–moderate–severe clusters recapitulate these same dimensions, indicating that the severity gradient identified in our unsupervised analysis reflects core PIRD pathobiology rather than cohort-specific patterns (34–36). This concordance supports the robustness and biological relevance of the clustering approach used in our study.

At the immunophenotypic level, we also observed patterns that are highly consistent with the published PIRD literature. Prior cohorts of CTLA-4 haploinsufficiency, LRBA deficiency and related disorders have repeatedly reported a combination of impaired regulatory T-cell compartment and features of inflammatory T helper cell subsets such as Tfh cells (37, 38). Across our severity clusters, patients in the more severe clinical groups showed the greatest deviation in these same axes, with more pronounced abnormalities in memory B-cell subsets, and regulatory and inflammatory T-cell subsets, whereas the mild cluster displayed comparatively preserved immune profiles. Thus, our unsupervised immunophenotypic clustering mirrors the key cellular signatures described in monogenic PIRD cohorts and extends them to a genetically heterogeneous population, supporting the notion that similar pathobiological endophenotypes underlie clinically convergent immune dysregulation. Moreover, the observation that these immunophenotypic signatures recur in our genetically diverse cohort reinforces the concept that fundamental axes of immune dysregulation are shared across distinct PIRD conditions. Demonstrating that the same perturbations in regulatory and activated T-cell compartments emerge even outside single-gene disorders provides an important contribution to the broader PIRD field. It suggests that these cellular endophenotypes represent common downstream pathways of immune dysregulation, and therefore may serve as cross-disease biomarkers for patient stratification, disease monitoring, and therapeutic decision-making in heterogeneous immune dysregulation syndromes.

Integration of IDS with CDS classified patients into three clusters that aligned with underlying genetic etiologies. Severe clusters were predominant for STAT3-GOF, STAT1-GOF, and LRBA deficiency, while milder groups were associated with STAT3-LOF, HAX1, and ADA2. These genotype-phenotype associations demonstrate the translational potential of IDS as a biomarker connecting cellular alterations with clinical outcomes. Composite machine learning models combining IDS, ΔIDS, mean values of inflammatory and regulatory cells slightly improved grouping in mild and moderate cases, although severe phenotypes remained poorly resolved due to intrinsic heterogeneity. This limitation is consistent with prior experiences in predictive modeling for autoimmunity and primary immunodeficiencies, where severe disease reflects multiple convergent mechanisms (39, 40).

Methodologically, our approach follows the direction of modern biomarker development, which emphasizes not only the ability to discriminate but also calibration and real-world clinical value. In our study, decision curve analysis showed a modest net benefit, suggesting that balance-based metrics can realistically be incorporated into decision-support tools (25). Clinically, IDS offers several potential applications. It provides an objective, quantifiable measure of immune balance that may add complementary value to clinical parameters for monitoring disease progression or treatment response. Its core cellular elements (Tregs, Bregs, Th17-like, and activated CD8^+^ T cells) represent dynamic targets of immunomodulatory therapy. Agents such as CTLA4-Ig and JAK–STAT inhibitors, already used in PIRDs directly modulate these compartments (34, 41). Serial IDS monitoring could thus serve as a pharmacodynamic biomarker, indicating whether restoration of regulatory/inflammatory equilibrium accompanies clinical improvement. Integrating such cellular biomarkers into therapeutic trials could integrates molecular and clinical data, advancing precision immunology from static diagnosis to dynamic disease monitoring.

Nevertheless, several points should be considered when interpreting these findings. The cohort size was modest and derived from a single center, which may limit generalizability. In addition, the cross-sectional design and the absence of an independent external validation cohort do not allow assessment of longitudinal IDS dynamics and currently preclude immediate clinical implementation. Replication in independent IEI populations will therefore represent an important next step. Within this framework, we sought to strengthen the robustness of our findings through comprehensive internal validation, including cross-validation to assess discrimination between PIRD and non-PIRD IEI groups, sensitivity and specificity analyses, and feature importance assessments demonstrating biological plausibility, with a consistent contribution from Treg-associated markers. Moreover, unsupervised clustering approaches revealed coherent and reproducible immune patterns that were well aligned with the IDS framework, supporting the internal consistency of the proposed approach.

Taken together, these results demonstrate that immune dysregulation is characterized by disrupted regulatory networks, expansion of inflammatory subsets, and redistribution of cellular clusters. IDS reliably separated patients from controls and correlated with clinical severity. Extending this approach, a composite model integrating multiple immune parameters modestly improved discrimination for mild and moderate cases, though performance remained limited in severe disease, underscoring the need for additional molecular features to refine predictive accuracy. By distinguishing PIRD from non-PIRD phenotypes and correlating with clinical severity, IDS provides both mechanistic insight and translational potential.

Future directions should focus on incorporating IDS into prospective, longitudinal cohorts to determine its utility as a biomarker of therapeutic response and clinical outcome. Integration with multi-omics approaches may refine biomarker panels and reveal layered signatures of dysregulation (42–44). Embedding IDS-based frameworks into interventional trials will be critical to test whether restoration of immune balance can serve as a surrogate for clinical improvement. Embedding such quantitative immune balance metrics into clinical workflows may enable personalized disease monitoring and targeted therapeutic decision-making, paving the way toward precision immunology in the management of immune dysregulation syndromes.

## Data Availability

The original contributions presented in the study are included in the article/**Supplementary Material**. Further inquiries can be directed to the corresponding author.
