# Supplementary material for: Cellular signatures of immune dysregulation in inborn errors of immunity: development of a quantitative immune balance score
Source: Front Immunol. 2026 Mar 5;17:1735655. doi: 10.3389/fimmu.2026.1735655 (PMC12999435; doi:10.3389/fimmu.2026.1735655)
Supplement: Supplementary file 1 [file Presentation1.pdf]

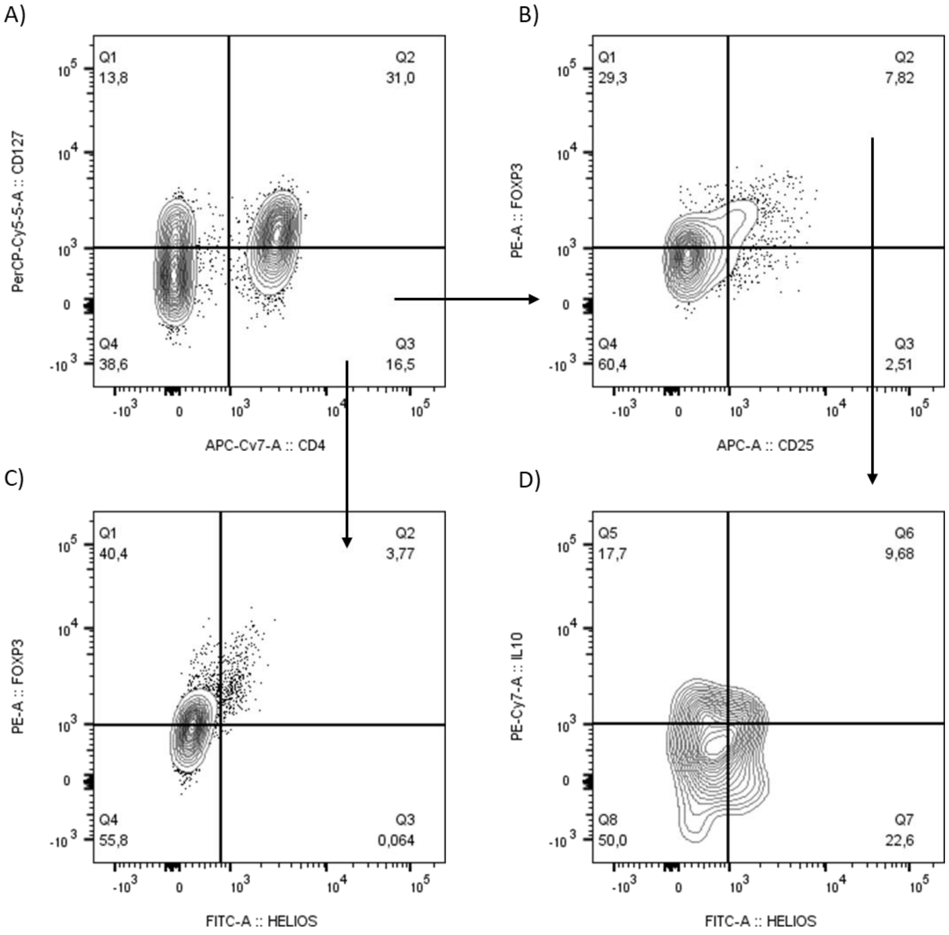

Sequential gating strategy for identification of Treg cells subsets.

(A) Lymphocytes were identified based on forward and side scatter characteristics (FSC-A vs SSC-A). $CD4^+$ and $CD127^{lo}$ T cells were gated from the lymphocyte population.

(B) Regulatory T cells (Tregs) were defined as $CD25^{hi}$ $FOXP3^+$ within $CD4^+$ and $CD127^{lo}$ T cells.

(C) $FOXP3^+$ Helios+ cells were defined within $CD4^+$ and $CD127^{lo}$ T cells.

(D) Functional subsets of Tregs were determined based on Helios and IL-10 expression, delineating $Helios^+$ and $IL\text{-}10^+$ populations.

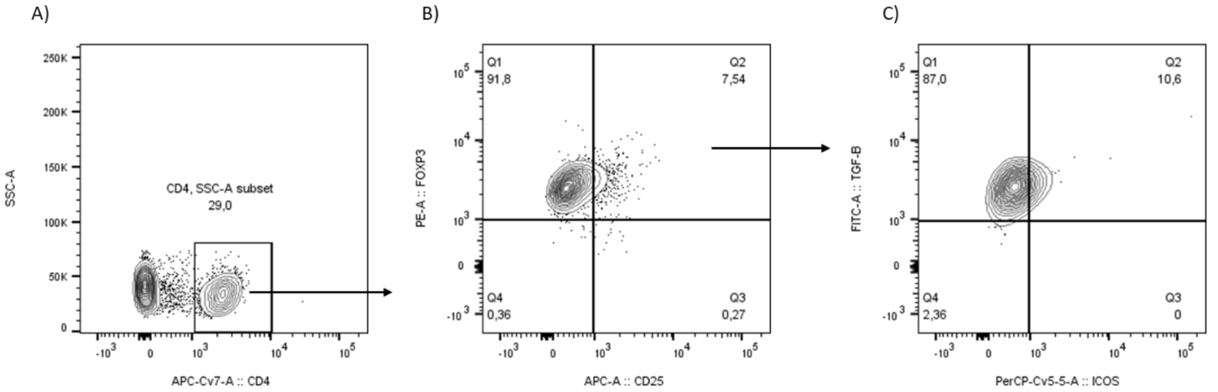

Sequential gating strategy for identification of Treg cell subset.

(A) Lymphocytes were identified based on forward and side scatter characteristics (FSC-A vs SSC-A). $CD4^{+}$ T cells were gated from the lymphocyte population.

(B) Regulatory T cells (Tregs) were defined as $CD25^{hi}$ $FOXP3^{+}$ within $CD4^{+}$ T cells.

(C) Functional subsets of Tregs were determined based on ICOS and TGF-β expression, delineating $ICOS^{+}$ and TGF-β^{+} populations.

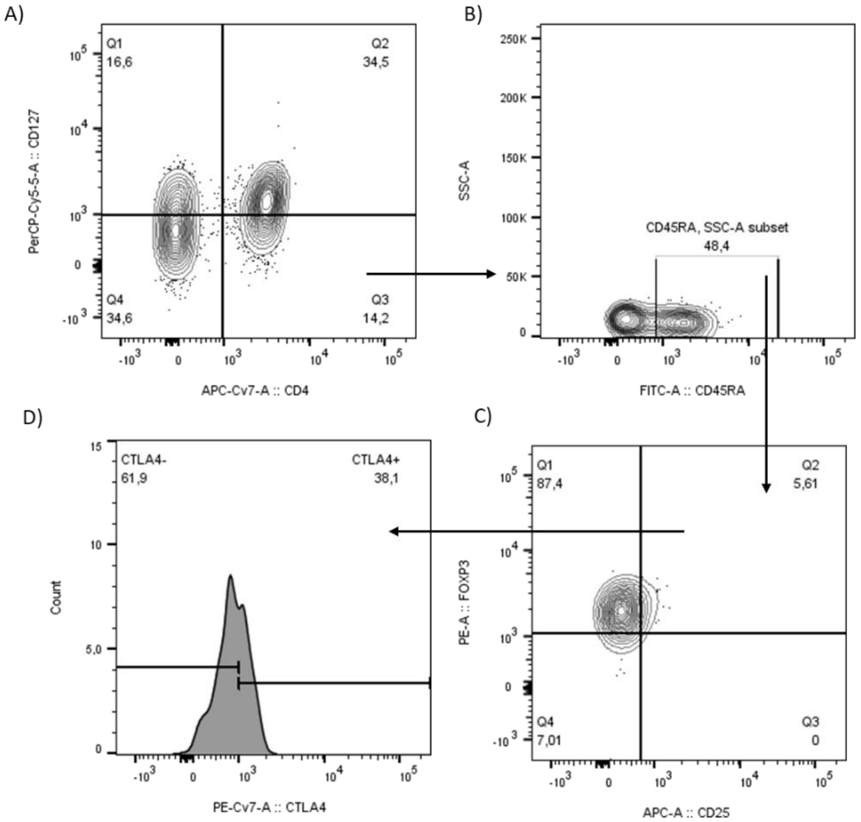

Sequential gating strategy for identification Treg cell subsets.

(A) Lymphocytes were identified based on forward and side scatter characteristics (FSC-A vs SSC-A). $CD4^{+}$ and $CD127^{lo}$ T cells were gated from the lymphocyte population.

(B)Naive regulatory T cells (Tregs) were defined as $CD45RA^{+}$ cells within $CD4^{+}$ and $CD127^{lo}$ T cells.

(C) $CD25^{hi}$ $FOXP3^{+}$ cells were defined within $CD4^{+}CD127^{lo}$ $CD45RA^{+}$ T cells.

(D) Functional subsets of these cells were determined based on CTLA4 expression.

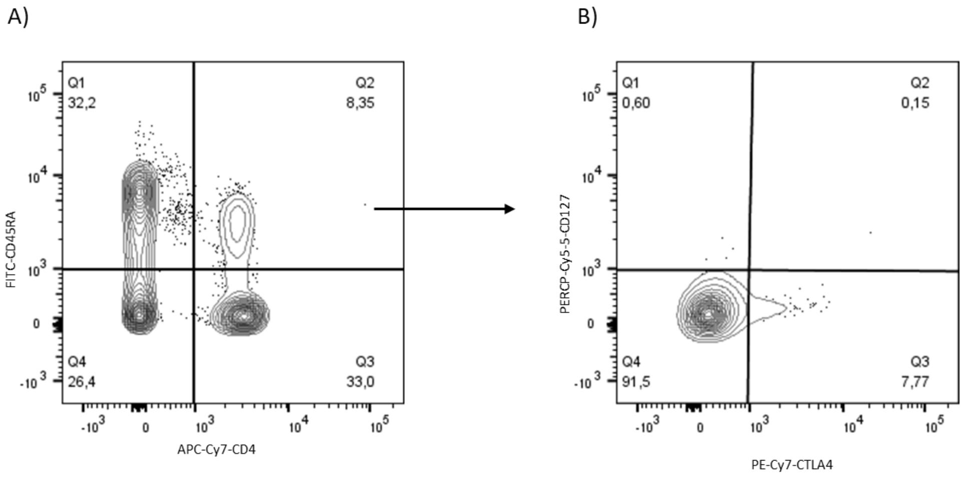

Sequential gating strategy of Treg cells subsets

(A) Peripheral blood mononuclear cells (PBMCs) were first gated on $CD4^+$ T cells based on CD45RA and CD4 expression.

(B) Within the $CD4^+$ $CD45RA^+$ cell subset, $CD127^{lo}$ $CD127^{hi}$ were analysed.

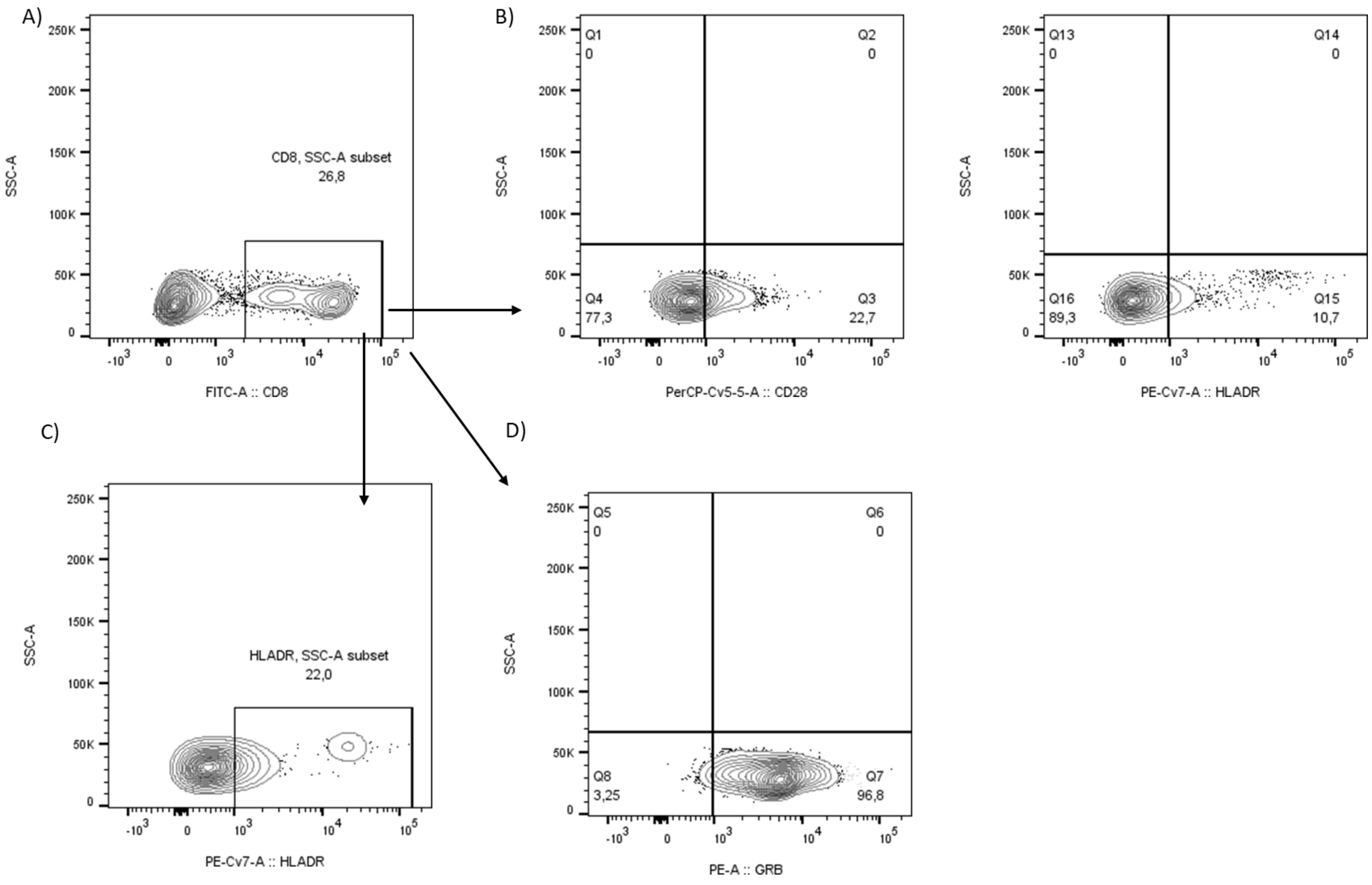

Sequential gating strategy for identification of CD8$^+$ T cell subsets.

(A) Lymphocytes were first selected according to side and forward scatter properties, and CD8$^+$ T cells were gated on the CD8 vs. SSC-A plot. (B) Representative density contour plots illustrate the hierarchical gating sequence, with the percentage of cells indicated within each gate for CD8$^+$CD28$^+$ HLA-DR$^+$ T cells. (C) Within CD8$^+$ population, expression of HLA-DR and (D) GrB was assessed to determine the frequency of activated CD8$^+$HLA-DR$^+$ T cells and CD8$^+$ GrB$^+$ T cells .

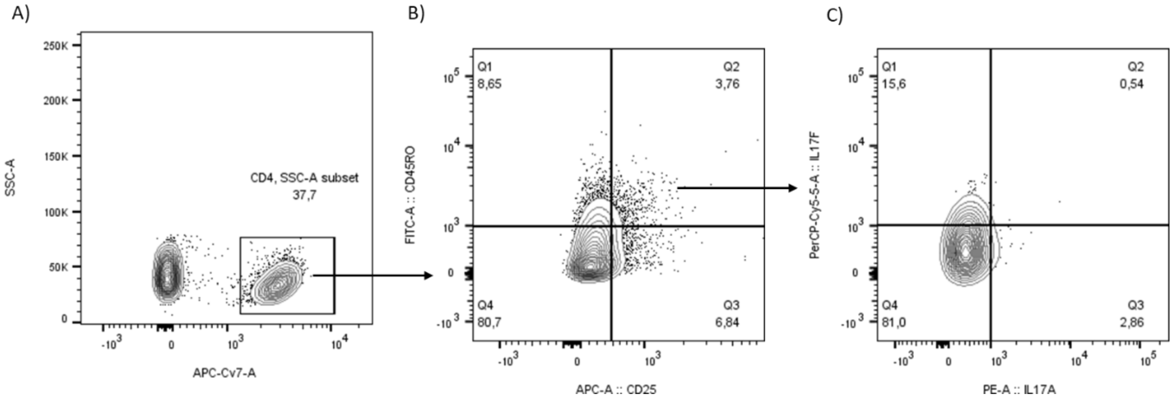

Sequential gating strategy for identification of Th17 cell subsets.
(A) Lymphocytes were selected according to forward and side scatter properties, and $CD4^+$ T cells were identified on the CD4 vs. SSC-A plot. (B) Within this population, expression of the $CD45RO^+$ and $CD25^+$ were assessed to define distinct functional subsets. (C) Representative contour plots illustrate the IL17A and IL17F distribution, with percentages indicating the frequency of positive cells within each quadrant.

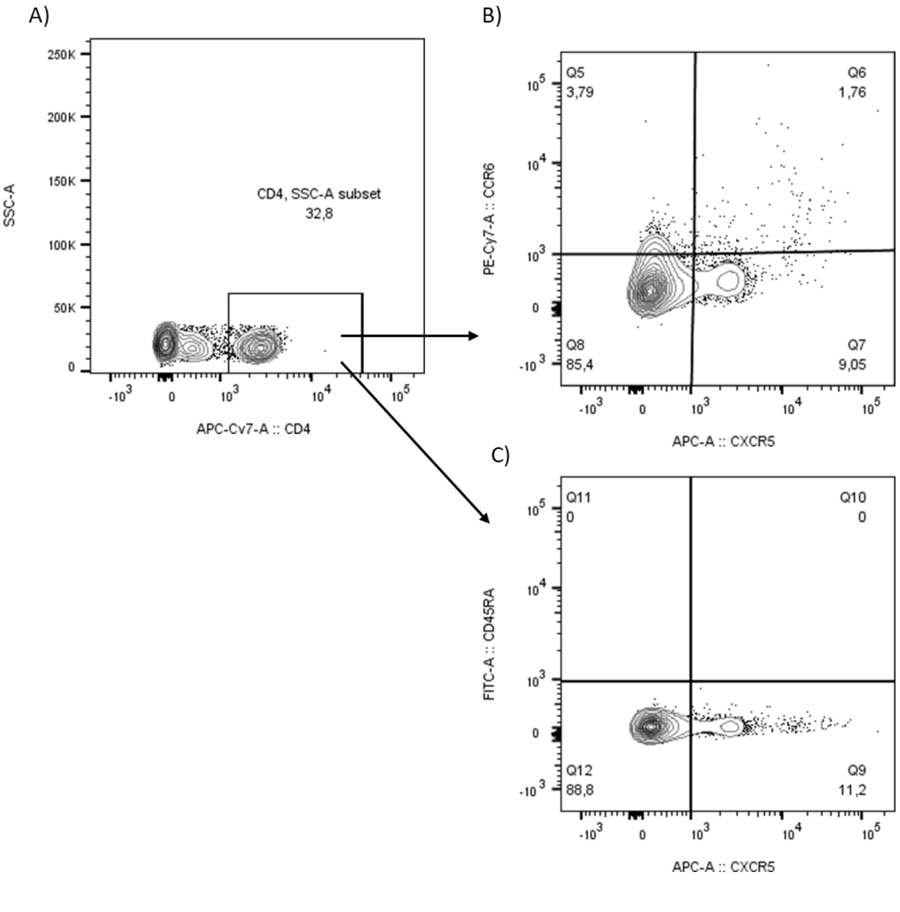

Sequential gating strategy for identification of cTFH cell subsets.
(A) Peripheral blood mononuclear cells (PBMCs) were first gated on $CD4^+$ T cells according to side scatter (SSC-A) and CD4 expression.
(B) Within the $CD4^+$ subset, CXCR5 and CCCR6 expression were analyzed to define the functional subsets of cTFH.
(C) CD45RA- CXCR5 cell subset was evaluated within the gated $CD4^+$ T cells.

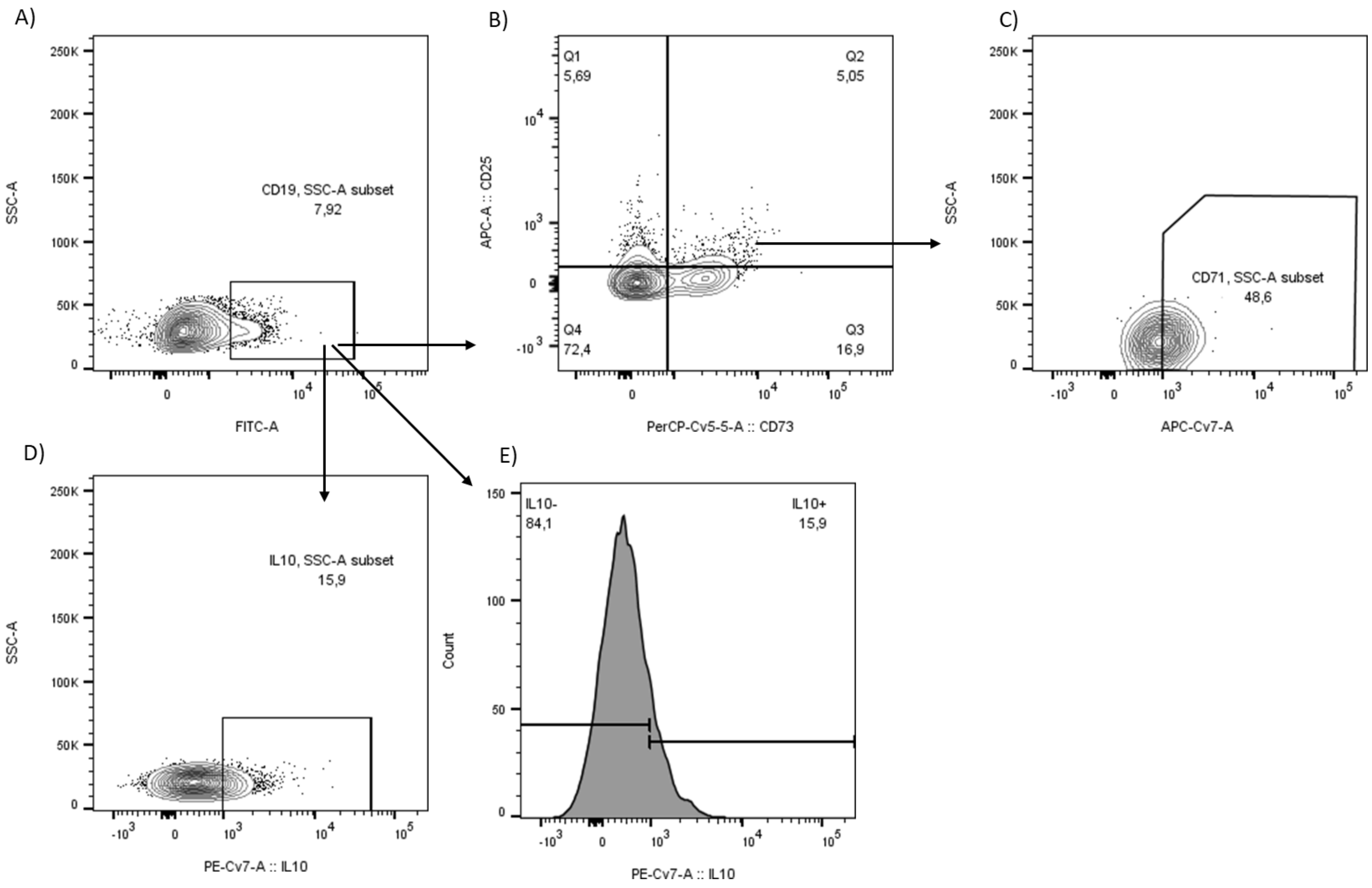

Sequential gating strategy for identification Breg cell subsets.

(A) Lymphocytes were first selected according to side and forward scatter properties, and CD19$^+$ B cells were gated on the CD19 vs. SSC-A plot. (B) Representative density contour plots illustrate the hierarchical gating sequence, with the percentage of cells indicated within each gate for CD25$^+$CD73$^+$ B cells. (C) Within this population, expression of CD71 was assessed. IL10 expression within CD19$^+$ cells was shown in (D) dot plot and (E) histogram plot.

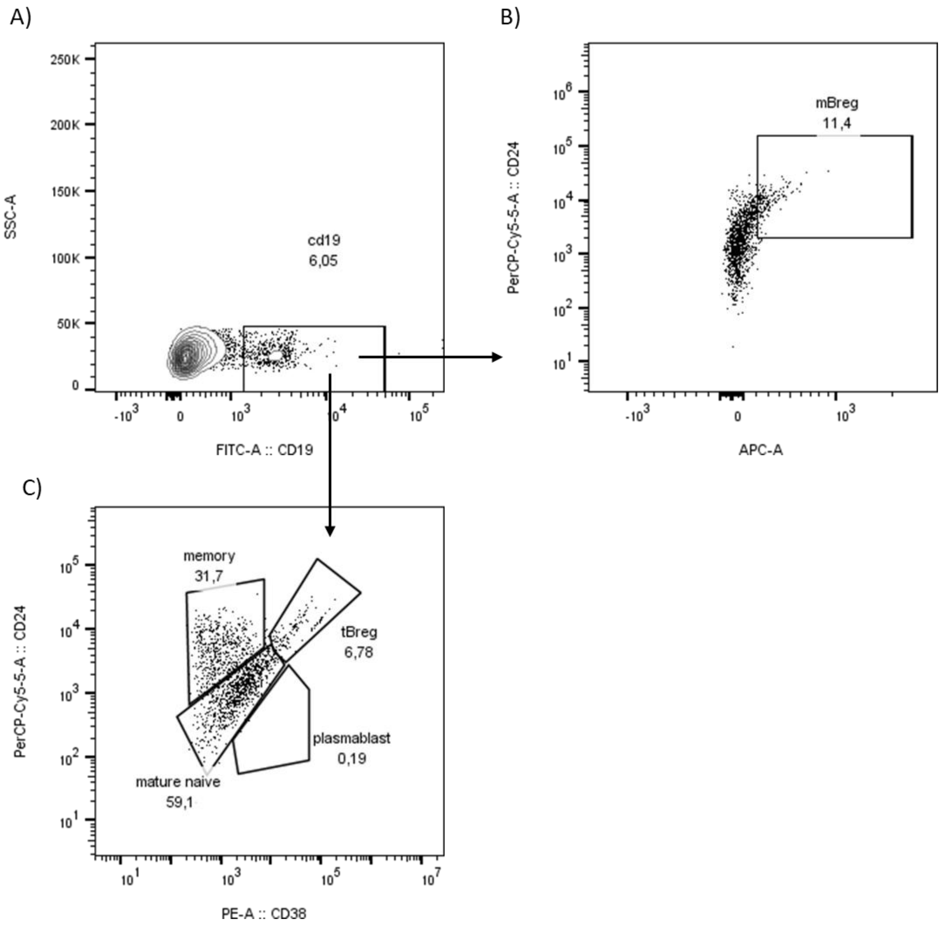

Sequential gating strategy for identification B cell subsets.
(A) Lymphocytes were first selected according to side and forward scatter properties, and $CD19^{+}$ B cells were gated on the CD19 vs. SSC-A plot. (B) Representative density contour plots illustrate $CD24^{+}$ and $CD27^{hi}$ mBreg cells. (C) Within $CD19^{+}$ B population, functional B cell subsets were evaluated due to expression level of CD24 and CD38.
