# Supplementary material for: Cellular signatures of immune dysregulation in inborn errors of immunity: development of a quantitative immune balance score
Source: Front Immunol. 2026 Mar 5;17:1735655. doi: 10.3389/fimmu.2026.1735655 (PMC12999435; doi:10.3389/fimmu.2026.1735655)
Supplement: Supplementary file 3 [file Table2.docx]

**Supplementary Table 2.** Antibody clones, fluorochromes, and flow cytometry panels used in this study

| **SPECIFITY** | **CLONE** | **FLUOROCHROME** | **SUPPLIER** | **PANEL** |
| --- | --- | --- | --- | --- |
| **CD4** | SK3 | APC-C7 | BD | CD4^+^ Treg subset |
| **CD25** | M-A251 | APC | BD | CD4^+^ Treg subset |
| **CD127** | HIL-7R-M21 | PERCP5.5cy | BD | CD4^+^ Treg subset |
| **CD45RA** | L48 | FITC | BD | CD4^+^ Treg subset |
| **CTLA4** | L3D10 | PE/Cyanine7 | Biolegend | CD4^+^ Treg subset |
| **FOXP3** | 259D/C7 | PE | BD | CD4^+^ Treg subset |
| **TGFB** | 21C11 | Purified | Biolegend | CD4^+^ Treg subset |
| **Helios** | 22F6 | FITC | Biolegend | CD4^+^ Treg subset |
| **CD8** | HIT8a | FITC | BD | CD8^+^ Treg subset |
| **Granzym B** | GB11 | PE | BD | CD8^+^ Treg and inflammatory subset |
| **CD28** | L293 | PERCP5.5cy | BD | CD8^+^ Treg subset |
| **HLADR** | G46-6 (also L243) | PE/Cyanine7 | BD | CD8^+^ Treg subset |
| **CD19** | SJ25C1 | FITC | BD | CD19^+^ Breg subset |
| **CD71** | CY1G4 | APC-C7 | Biolegend | CD19^+^ Breg subset |
| **CD73** | AD2 | PERCP5.5cy | BD | CD19^+^ Breg subset |
| **CD19** | HIB19 | FITC | BD | CD19^+^ Breg subset |
| **IL10** | JES3-9D7 | PE/Cyanine7 | Biolegend | CD19^+^ Breg subset |
| **CD38** | HB7 | PE | BD | CD19^+^ Breg and inflammatory subsets |
| **CD24** | ML5 | PERCP5.5cy | BD | CD19^+^ Breg and inflammatory subsets |
| **CD27** | M-T271 | APC | BD | CD19^+^ Breg and inflammatory subsets |
| **CXCR5** | RF8B2 | APC | BD | CD4^+^ cTFH subset |
| **CCR6** | 11A9 | PE/Cyanine7 | BD | CD4^+^ cTFH subset |
| **PD-1** | A17188B | PE | Biolegend | CD4^+^ cTFH subset |
| **BCL6** | K112-91 | PE/Cyanine7 | BD | CD4^+^ cTFH subset |
| **ICOS** | DX29 | PERCP5.5cy | BD | CD4^+^ cTFH subset |
| **IL17A** | N49-653 | PE | BD | CD4^+^ Th17 subset |
| **IL17F** | O33-782 | PERCP5.5cy | BD | CD4^+^ Th17 subset |
| **CD45RO** | UCHL1 | FITC | BD | CD4^+^ Th17 subset |
