# Supplementary material for: Cellular signatures of immune dysregulation in inborn errors of immunity: development of a quantitative immune balance score
Source: Front Immunol. 2026 Mar 5;17:1735655. doi: 10.3389/fimmu.2026.1735655 (PMC12999435; doi:10.3389/fimmu.2026.1735655)
Supplement: Supplementary file 4 [file Table3.docx]

# Supplementary Table 3. Overview of the Immune Dysregulation Score (IDS)

| **Section** | **Item** | **Description** |
| --- | --- | --- |
| **Inputs** | Regulatory cells | CD4^+^ Treg subsets  CD8^+^ Treg subsets  CD19^+^ Breg subsets |
|  | Inflammatory markers | cTFH cell subsets  Th17 cell subsets  CD8^+^ cell subsets  CD19^+^ cell subsets |
|  | Source & assay | Peripheral blood; multiparametric flow cytometry; standardized compensation/QC procedures |
| **Computation** | Base formula | IDS = log₂ [(Inflammatory_mean)/(Regulatory_mean)] |
|  | Scaling/normalization | Z-score normalization relative to healthy controls; batch correction (ComBat/Harmony) |
|  | Feature weighting | FlowSOM metacluster-based z-scores (MC2, MC4, MC5) used as feature weights |
| **Performance** | Discrimination | ROC/AUC (patients vs controls); multiclass AUC for severity subgroups |
|  | Calibration | Decile-binned calibration curve; bootstrap validation; slope/intercept reporting |
|  | Clinical utility | Decision curve analysis showing net clinical benefit across risk thresholds |
| **Interpretation** | IDS ranges | Low (≤ 0): regulatory-dominant; Intermediate (0–0.7); High (≥ 0.7): inflammatory-dominant |
|  | Clinical mapping | Low → infection-predominant mild; Intermediate → mixed; High → autoimmunity/lymphoproliferation severe |
|  | Genotype patterns | STAT1/3-GOF & LRBA → high IDS; STAT3-LOF/HAX1/ADA2 → low/intermediate |
|  |  |  |
|  |  |  |
| **Limitations** | Design | Single-center, cross-sectional |
|  |  |  |
| **Reporting** | Reproducibility | Antibody clone/fluor table **(Supplementary Table 2**)  Gating strategy **(Supplementary Figure 1)** |
